# Identification of Peptide Mimics of a Glycan Epitope on the Surface of Parasitic Nematode Larvae

**DOI:** 10.1371/journal.pone.0162016

**Published:** 2016-08-31

**Authors:** Saleh Umair, Qing Deng, Joanna M. Roberts, Richard J. Shaw, Ian A. Sutherland, Anton Pernthaner

**Affiliations:** 1 AgResearch Limited, The Hopkirk Research Institute, Palmerston North, New Zealand; 2 Current affiliation: Flowjoanna Tapui Limited, No.1 Line, Longburn, Palmerston North, New Zealand; Universidade Federal de Minas Gerais, BRAZIL

## Abstract

Phage display was used to identify peptide mimics of an immunologically protective nematode glycan (CarLA) by screening a constrained C7C peptide library for ligands that bound to an anti-CarLA mAb (PAB1). Characterisation of these peptide mimotopes revealed functional similarities with an epitope that is defined by PAB1. Mimotope vaccinations of mice with three selected individual phage clones facilitated the induction of antibody responses that recognised the purified, native CarLA molecule which was obtained from *Trichostrongylus colubriformis*. Furthermore, these mimotopes are specifically recognised by antibodies in the saliva of animals that were immune to natural polygeneric nematode challenge. This shows that antibodies to the PAB1 epitope form part of the mucosal polyclonal anti-CarLA antibody response of nematode immune host animals. This demonstrates that the selected peptide mimotopes are of biological relevance. These peptides are the first to mimic the PAB1 epitope of CarLA, a defined larval glycan epitope which is conserved between many nematode species.

## Introduction

The nematode glycan CarLA (carbohydrate larval antigen) is expressed in the cuticle of parasite 3^rd^ stage larvae (L3) of numerous species capable of infecting livestock [1]. Preliminary investigations of the purified molecule established it to be a glycolipid which can be purified by hot water extraction. The molecule is reasonably resilient to acid/base degradation and is free of any protein component [1].

Immunologically, CarLA is a protective antigen in sheep and potentially in other livestock species [2]. The mucosal antibody response to the molecule consists predominantly of IgA and IgG and prevents establishment of ingested larvae. While the detailed mechanism is unclear, it involves reducing motility or induction of clumping of larvae [1]. Furthermore, the mucosal polyclonal anti-CarLA IgA antibody response has been found to be reflective of the immune status of the animal, as measured by faecal egg counts (FEC) [3], and is also correlated with the level of larval challenge. The molecule is conserved between many nematode species, although epitopic variation of some of the protective surface epitopes between and within individual nematode species has been described [4]. The antigen is present on the surface of exsheathed L3 but not present following development into the L4 stage [5]. Some of these immunologically-relevant features make CarLA a promising vaccine candidate if sufficient amounts of the native molecule or a synthesised product could be obtained. However, to date the structure and detailed molecular composition of CarLA have not been defined and chemical synthesis of epitopes or the entire molecule remains unlikely to be achieved in the near future.

The mAb PAB1 recognises an epitope of CarLA which is conserved between many nematode species, suggesting functional importance [6] although the detailed immunological properties of the PAB1 epitope are not yet known. While the PAB1 epitope is not expressed on the surface of infective larvae, reactivity is evident against the purified glycan or against exsheathed L3 stage larvae following heat treatment. It remains to be determined if antibodies to this epitope form part of the polyclonal anti-CarLA antibody response of host animals which are demonstrably immune to nematode challenge.

Purification of native CarLA is a tedious process requiring the culturing of millions of L3 from large amounts of animal faeces, exsheathing the L3s and separating from sheaths before extraction of the CarLA molecule. This limits the ability to elucidate further some of the functional roles and structure of CarLA. The aim of this study, therefore, was to identify a peptide mimic of the glycan PAB1 epitope which shares conformational and immunological properties of the parent molecule, and which could serve as a functional substitute for the nematode glycan. Such an approach has been successfully employed by others to develop peptide mimotopes that immunologically mimic carbohydrate antigens expressed on bacteria, viruses and cancer cells [6].

## Material and Methods

### Parasitology, isolation of CarLA and sampling of saliva

Sheep experiments complied with the New Zealand Animal Welfare Act and were approved by the Grasslands Animal Ethics Committee (AEC 11845). Lambs were housed indoors in the Grasslands Animal facility and experimentally infected with L3 stage larvae of the intestinal nematode parasite *Trichostrongylus colubriformis*. No detrimental effects to health and wellbeing of lambs were observed. At the end of the experiment, lambs were drenched and returned to the farm. L3 larvae were then cultured from faeces collected of these lambs. Purified larvae were exsheathed in sodium hypochlorite and the nematode glycan CarLA isolated as a hot water extract followed by column purification as described previously [1].

Pools of saliva samples either positive or negative for a polyclonal anti-CarLA salivary IgA and IgG response were prepared. The positive pool consisted of 57 samples from naturally infected sheep that were selected from routine samples collected by farmers for analysis of the resistance status. The positive pool was likely to contain antibodies with specificity for the PAB1 epitope on CarLA. The 35 saliva samples that compiled the negative pool were obtained from field samples collected on an AgResearch research farm (AEC 12821).

### Peptide library screening

A disulphide-constrained heptapeptide phage display library with a complexity of 1.2 x 10^9^ transformants (Ph.D.-C7C^TM^ peptide library, New England Biolabs, Cambridge, MA, USA) was used to select for phage with specific affinity for PAB1.

#### Pre-panning procedures

The mAb PAB1 reacts with a CarLA-specific epitope, which is conserved between many parasitic nematode species [6]. PAB1 was produced in house. The mAb was purified from overgrown culture supernatant by absorption and elution from a Protein G Superose [7]. Purified PAB1 mAb was then diluted into coupling buffer for conjugation to beads as outlined below.

PAB1-coated beads were prepared using carboxyl (COOH) micro particles (Bangs Laboratories) in conjunction with the PolyLink Protein Coupling Kit (Bangs Laboratories). Briefly, micro particles were resuspended in PolyLink Coupling Buffer and carbodiimide solution prior to the addition of 500μg PAB1. Following incubation for 1 hour beads were washed before storing at 4°C until use.

#### Panning procedure

Three rounds of biopanning were performed using affinity purified PAB1 immobilised to beads in a protocol similar to that recommended by the manufacturer of the library. Before panning, free binding sites of PAB1-coated beads were blocked by incubation with 0.2% Polyvinylpyrrolidone (PVP) in PBS supplemented with 0.05% Tw20 (PBS/Tw20) for 2 h at room temperature (RT). An ER2738 culture was prepared by overnight in 2xYT/Tet (20) at 37°C which was then diluted 1:50 in 2xYT and grown at 37°C with shaking until an OD of 0.5 was reached. An aliquot of Ph.D.-C7C^TM^ peptide library (or 1^st^ panning amplified phage eluate or 2^nd^ panning amplified phage eluate) was added to the beads and incubated under gentle agitation for 30 min, followed by incubation without agitation for a further 90 min at RT. Unbound phage was then removed by washing beads extensively 8 times with PBS/Tw20 followed by 8 washes in PBS. PAB1 beads with bound phage were then added to the ER2738 culture, incubated at 37°C under slow shaking for 30 min followed by vigorous shaking for an additional 4.5hr at 37°C. An aliquot was then removed to establish elution phage titres before phage amplification. The culture was spun at 8000rpm for 10mins, at 4°C. Phage was then precipitated from the supernatant in a 1/5 volume of 20% PEG/2.5M NaCl at 4°C O/N. The PEG precipitate was spun at 10000rpm for 15mins at 4°C. The pellet was re-suspended in PBS and spun for 5mins at 4°C to remove residual cells. The supernatant was re-precipitated with a 1/5 volume of PEG/2.5M NaCl, by incubation at 4°C for 4h followed by centrifugation at 14000 rpm for 10mins at 4°C. The pellet was suspended in PBS and titrated along with the unamplified eluate.

The titre of the unamplified eluate and amplified eluate was determined by diluting 10 μl of phage with 200μl of mid-log phase ER2738, incubation at RT for 5min and addition of 3ml of pre-warmed 0.7% soft agar onto 2xYT/ IPTG/ Xgal plate and ON incubation at 37°C. For the second and third round of biopanning, the stringency of washings was enhanced by increasing the concentration of detergent in the wash buffer from 0.05 to 0.2% Tw20.

### Isolation and characterisation of individual phage clones

48 individual phage clones were established by amplifying library phage from individual plaques of the titre plates from the third biopanning in *E*. *coli* for 4.5 h. Bacteria were then removed by centrifugation, and the phage-containing supernatant stored at 4°C until used for isolation of DNA.

Single stranded phage DNA was purified from phage containing culture supernatant by QIAprep spin column purification as recommended by the manufacturer (Qiagen, Hilden, Germany). Quantity and purity of purified DNA was then measured on a Nanodrop spectrophotometer (Thermo Scientific, Asheville, NC). Single-stranded DNA was sequenced (Waikato DNA Sequencing Facility, Hamilton, New Zealand) using the 96gIII sequencing primer and the nucleotide sequence of the gIII insert determined and translated into a peptide sequence as recommended (New England Biolabs).

Purified phage clones were then tested for their ability to bind the immobilised PAB1 mAb. ELISA plates (Maxisorp, Nunc, Denmark) were coated with 2 μg/ml purified PAB1 in PBS overnight at 4°C, washed with PBS and then blocked with PVP as above for beads. Plates were then washed again with PBS, incubated with a 1:20 dilution of purified phage in blocking buffer for 1.5h, and then extensively washed as above for beads. Bound phage was then detected via an HRP-conjugated antiM13 mAb (GE Healthcare, Little Chalfont, UK) and colour developed with Tetramethylbenzidine (TMB).

### Peptide immunoassays

Peptides that were repeatedly identified in the elution of the third biopanning were synthesised in high purity by Apeptide (Shanghai, China) or by JPT (Berlin, Germany) as disulphide bonded, biotinylated molecules. A Lys (PEG3) spacer was introduced to physically separate biotin from peptide to reduce the potential effect of steric hindrance.

The peptides were dissolved in a small volume of required solvent (DMF or acetic acid), diluted in PBS and then immobilised on ELISA plates (Maxisorp NUNC, Denmark) at 4°C and overnight incubation. Concentrations ranged from 0.1 to 5 μg/ml depending on the specific aims of the experiments. Plates were than washed 3-times in PBS and free binding sites were then blocked with SuperBlock Blocking Buffer (Thermo Scientific) by incubation at room temperature for 30 min. Plates were then washed 3-times in PBS supplemented with 0.1% Tw20 (PBS/Tw20) followed by 3 washes with PBS. Dilutions of the saliva pools in ELISA buffer were then added and incubated for 2 h at 37°C. The range of dilutions used is detailed below in the protocols for the experiments. Plates were then washed 3-times in PBS/Tw20 and 3-times in PBS. Specific binding of salivary antibodies was then detected with a rabbit anti-sheep IgA-HRP (Bethyl Laboratories, Montgomery, Texas; diluted 1:4000 in ELISA buffer)—or rabbit anti-sheep IgG-HRP conjugated antibody (DAKO, Denmark; diluted 1:5000 in ELISA buffer) by incubation for 2 h at 37°C. Plates were then rewashed as previously and colour developed with TMB as described previously [3,8].

### CarLA absorption ELISA

Absorption ELISAs were designed to determine if the antibody recognition of peptide mimotopes of CarLA in saliva is inherent to the overall saliva anti-CarLA response. CarLA (5 μg/ml) was immobilised to the wells of ELISA plates by overnight incubation at 4°C. Plates were then incubated with two-fold serial dilutions of pooled saliva in ELISA buffer ranging from 1:10 to 1:1280 for 2 h at 37°C followed by incubation at 4°C overnight. In some of the experiments this absorption procedure was repeated a second time. The pre-absorbed saliva pool dilutions were then assessed for IgA and IgG as described in section 2.5 for peptides.

### Peptide absorption ELISA

Individual peptides were immobilised to the wells of ELISA plates at 2 μg/ml as above. Plates were then washed, blocked and incubated with the CarLA pre-absorbed saliva pool as for the CarLA absorption ELISA. The pre-absorbed saliva was then assayed for reactivity against either the peptide used for absorption, or against CarLA. Specific IgA and IgG binding was measured as above.

### Competition ELISAs

Synthetic peptide PAB1.C7C-16, or a pool of 5 peptides (PAB1.C7C-4, -5, -16, -18, -37), was tested for the ability to compete with mAb PAB1 for binding to immobilised CarLA. Plates were coated with CarLA as above, and then incubated with two-fold serial dilutions of peptide ranging from 20 to 0.3125 μg/ml in the presence of a fixed amount of PAB1 (a 1:400 dilution of an overgrown hybridoma culture supernatant). In another set of assays, individual peptides PAB1-C7C-3, -4, -5, -16, -17, -18 and -37 were tested at 10, 5 and 2.5 μg/ml for the ability to compete with polyclonal antibodies in the CarLA positive saliva pool (diluted 1:20) for binding to immobilised CarLA. For both sets of assays a non-selected C7C peptide (at 10, 5 and 2.5 μg /ml) and purified CarLA (at concentrations ranging from 2 to 0.03125 μg/ml) served as negative and positive controls, respectively.

### Immunisations and serum antibody assays

Animal experiments complied with the New Zealand Animal Welfare Act and were approved by the Grasslands Animal Ethics Committee (AEC 12940). Groups of 2–3 months old male BALB/c mice (n = 3) were housed in the Grasslands small animal facility and immunised at weeks 0 and 2 with approximately 6x10^11^ pfu of the phage clones PAB1.C7C-3, PAB1.C7C-4 or PAB1.C7C-16. Phage were re-suspended in 200 μl PBS and injected intra-peritoneally. Blood was obtained at weeks 0, 2 and 4 by tail pricks and serum samples were stored at -20°C until analysis. All mice were euthanised at the end of trial using CO_2_ anaesthesia followed by cervical dislocation as per ethics committee requirements. Mice were active and healthy during the course of the trial and no animal died prior to the trial end point.

Serum samples were tested by ELISA for the presence of antibodies that reacted with the purified native parasite antigen CarLA. Plates were coated with 5 μg/ml CarLA, blocked and washed as above and then incubated with serum diluted 1:800 in ELISA buffer for 2 h at RT. Bound immunoglobulins were then detected by incubation with rabbit anti-mouse Ig-HRP (diluted 1:1000) for 2 h at 37°C and colour developed as above.

### Statistical analysis and bioinformatics

A mixed effects model with fixed effects treatment, time and their interaction and random effect was used to analyze natural log transformed response OD in the pre-absorption of saliva and the vaccination studies. The significance level was set as P < 0.05. A multiple comparison of the interested means was performed with p-value adjusted by 'BH' method. The analysis was produced by packages 'nlme' and 'predicted means' in R. Geneious 8 (Biomatters Ltd) was used for alignment of the sequences and blast searches.

## Results

### Biopanning and peptide sequence analysis

Biopanning of the constrained C7C peptide library for binding to the mAb PAB1, which exhibits specificity for the nematode glycan antigen CarLA, resulted in enrichment for library phage clones during the selection process. The percentage of phage enrichment after three rounds of biopanning are presented in Table 1. Although, roughly the same amount of PAB1 antibody the phage input for the three rounds of panning, the specific phages bound to PAB1 increased. The eluted phage yield (%) from the first to the third panning increased from 1.1 x 10^−5^ to 5.1 x 10^−3^, an enrichment is about 460 times. The data demonstrated a successful affinity selection of phage that were ligands for PAB1 mAb.

**Table 1:** Enrichment of phage (% yield) by biopanning.

Biopanning	Phages added	Phages eluted	Yield (%)
1	2x10^11^	2.2x10^6^⁶	1.1x10^-5^
2	2x10^11^	7x10^7^	3.5x10^-4^
3	1.5x10^11^	7.7x10^8^	5.1x10^-3^

Nucleotide sequence analysis of the gIII insert resulted in the correct identification of 45 out of 48 individual phage clones, with the remaining three not containing an insert. Sequence analysis of the translated nucleotide sequences resulted in the identification of 12 unique peptides. Seven of these were repeatedly identified, with a frequency ranging from 2 to 14 times (Table 2). This provided evidence for selective enrichment by biopanning for library phage clones with specific binding properties for the PAB1 mAb.

**Table 2:** Deduced peptide sequences and frequency of identification after biopanning. Amino acid motifs present in 2 or more peptides are shown in bold.

Peptide ID	Peptide sequence	Frequency of identification
PAB1.C7C-36	CGEA**SSP**RC	1
PAB1.C7C-5	CGVHSQ**SS**C	9
PAB1.C7C-15	CI**TTP**GFLC	1
PAB1.C7C-4	CKN**PTT**GTC	14
PAB1.C7C-40	CKS**TP**TNGC	1
PAB1.C7C-16	CN**TP**MQRSC	3
PAB1.C7C-24	CP**PS**KYHLC	1
PAB1.C7C-7	CPQHFKTFC	1
PAB1.C7C-17	C**SL**RGHDLC	2
PAB1.C7C-3	CS**SSP**YAWC	6
PAB1.C7C-18	CTKHT**LS**IC	2
PAB1.C7C-37	CW**SS**FRDEC	4

Sequence analysis of the peptides did not reveal any putative conserved domains or exact matches with any known proteins as determined by BLAST search. Of note is that the selection process was dominated by three clusters of peptides with amino acid motifs TTP or TP, SSP, SS or PS, and SL, which were found in several of the identified peptides, albeit in different positions (Table 2). All of the identified peptides contained either serine or proline.

#### Selected phage clones bind to mAb PAB1

**Fig 1:**
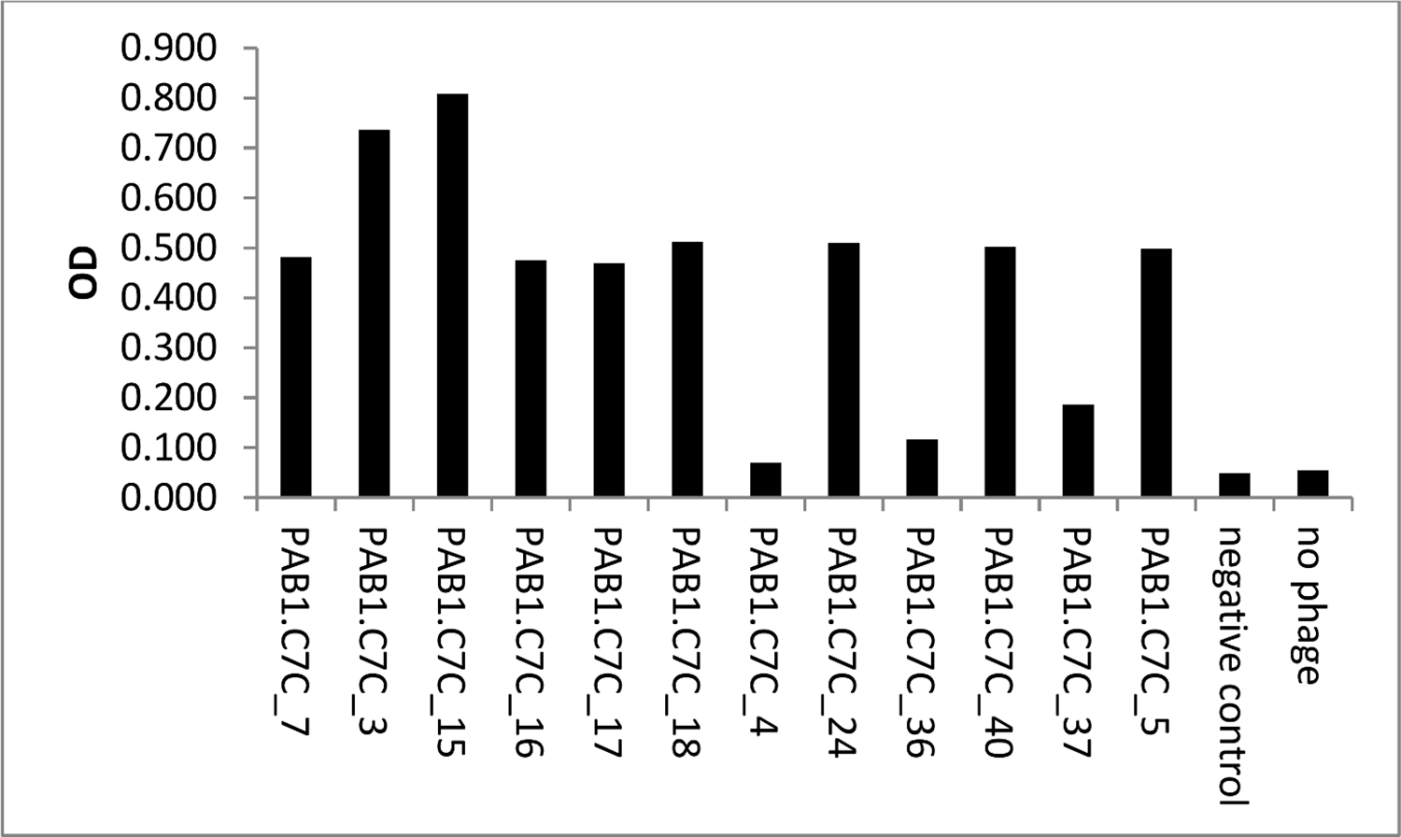
Biopanning results in the identification of phage clones that bind to mAb PAB1. Immobilised mAb PAB1 recognised a selection of purified phage clones diluted 1:20, negative control: VCSM13.

Phage clones for which peptide sequences were determined were then tested for their ability to bind to immobilised PAB1 (Fig 1). Binding to PAB1 was observed in 11 out of 12 phage clones (Fig 1); wild type phage (VCSM13 helper phage) were used as negative control and positive binding was considered when OD was > 0.1. This indicated the identification of potential mimotopes for the PAB1 CarLA epitope.

### Synthetic PAB1 mimotopes are recognised by IgA and IgG in saliva

**Fig 2:**
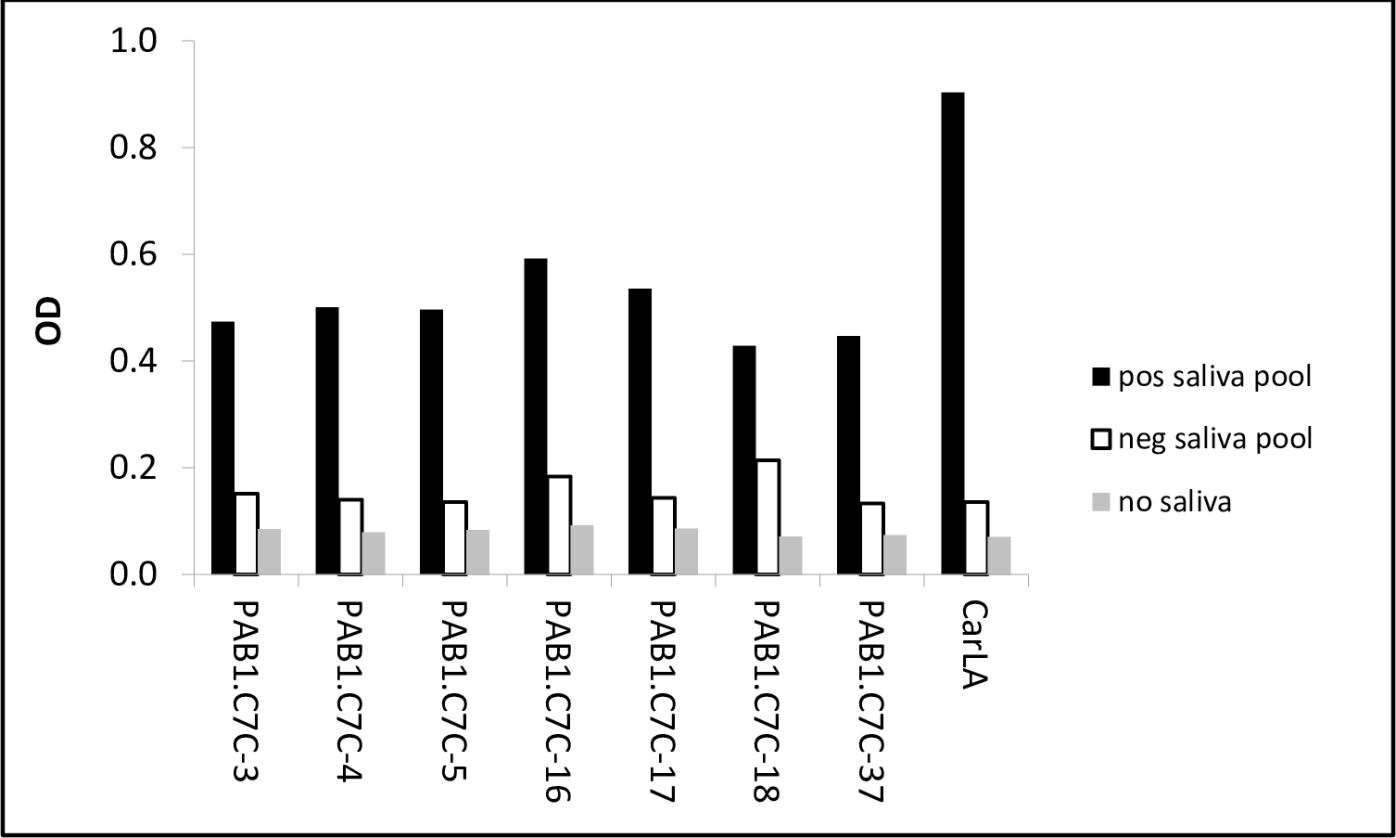
Recognition of peptides by salivary IgA. Plates were coated 2 μg/ml of individual peptides or with purified CarLA (2 μg/ml). A CarLA-negative saliva pool was used to test for non-specific binding. Peptides PAB1.C7C-3, -4, 5-, 16-, 18 and 37 were recognised by salivary IgA from an anti-CarLA positive pool. Serial dilutions of saliva pools were used in assays and results from 1:10 dilutions presented. Data are representative of at least 3 independent repeat assays.

**Fig 3:**
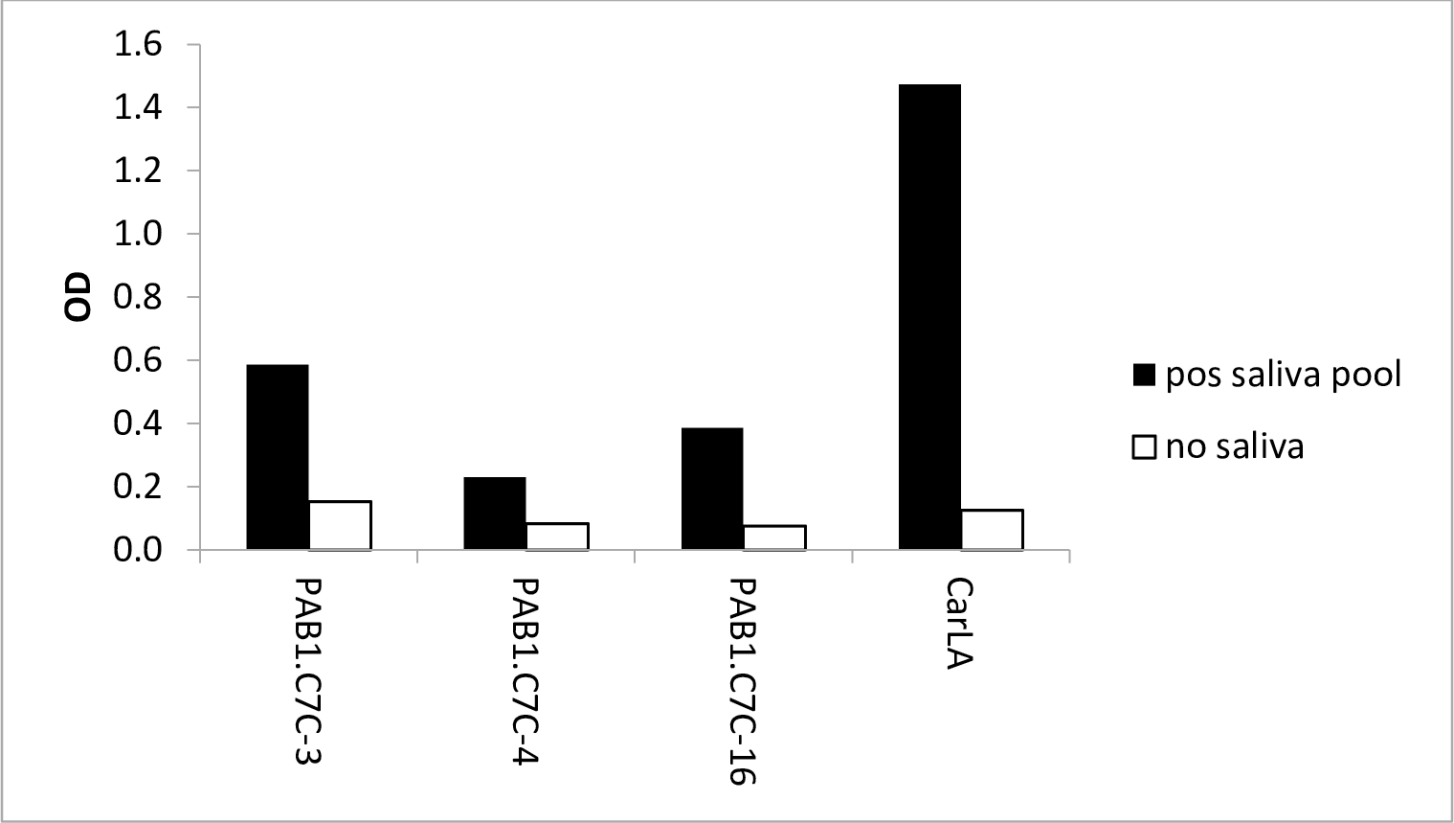
Recognition of peptides by salivary IgG. Plates were coated with 5 μg/ml of a selection of individual peptides or with purified CarLA (2 μg/ml). Saliva diluent only was used to test for non-specific binding. Peptides PAB1.C7C-3, -4 and -16 were recognised by salivary IgG from an anti-CarLA positive pool. Serial dilutions of saliva pools were used in assays and results from 1:10 dilutions presented. Data are representative of two independent experiments.

We next investigated if some of the identified peptides mimic the PAB1 epitope on native CarLA. Their potential function as mimotopes was evaluated by testing polyclonal anti-CarLA antibodies for reactivity with the synthetic peptides. We utilised a saliva pool which was collected from sheep that showed a strong polyclonal anti-CarLA antibody response, and therefore was likely to contain antibodies with specificity for the PAB1 epitope. We found that a selection of these peptides (PAB1.C7C-3, 4, -5, -16, -17, -18 and -37) was recognised by IgA in saliva (Fig 2), providing additional evidence for the identification of biologically relevant mimotopes. Furthermore, peptides PAB1.C7C-3, -4 and -16 were also recognised by antibodies of the IgG subclass from an anti-CarLA positive saliva pool (Fig 3). This demonstrates that IgA and IgG in saliva, which exhibits a strong polyclonal reactivity to the nematode larval antigen CarLA, specifically bound to some of the identified mimotopes.

### Pre-absorption of saliva with synthetic peptides or CarLA reduces reactivity to mimotope

**Fig 4:**
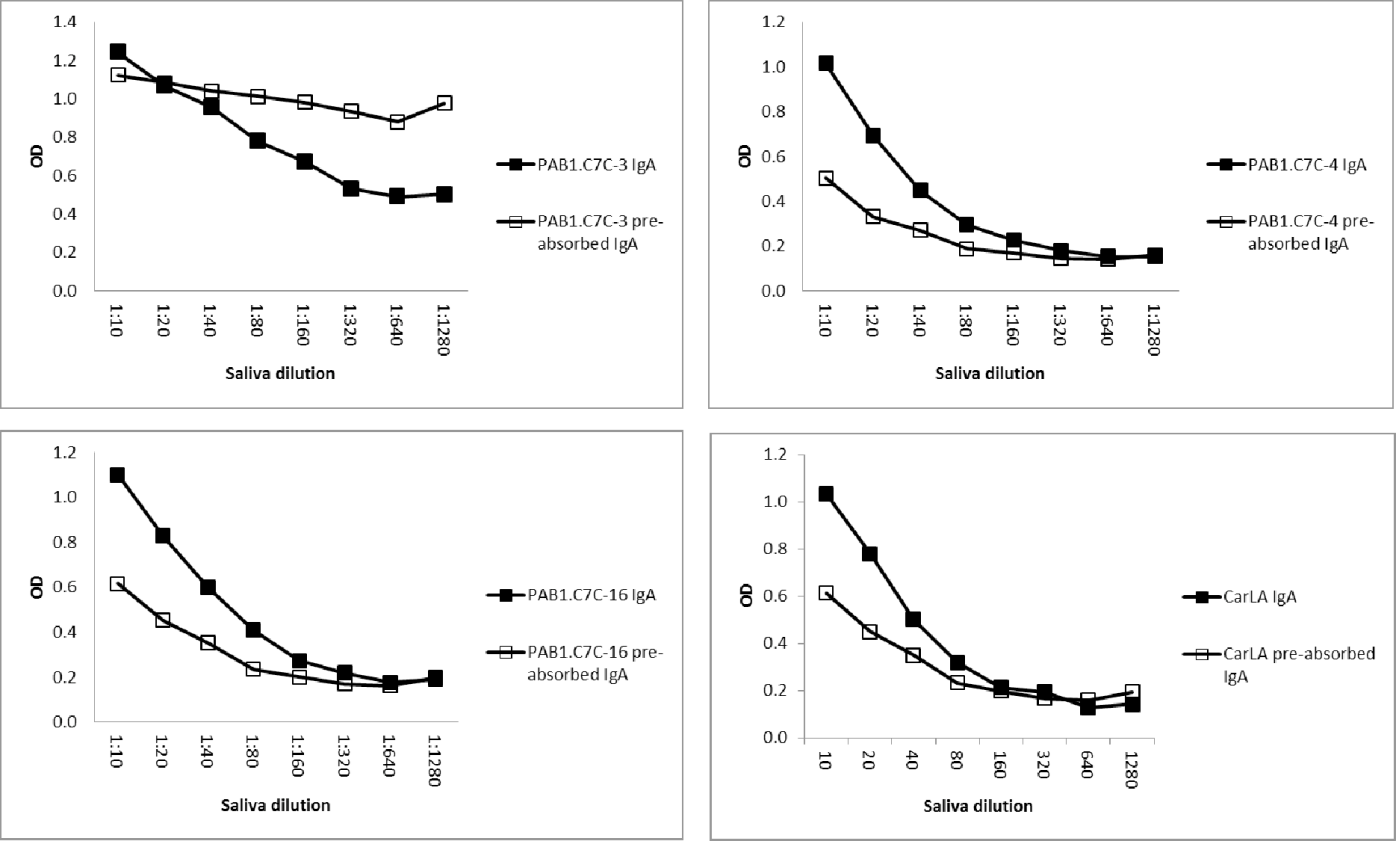
Pre-absorption of saliva with peptides resulted in selective depletion of peptide specific IgA. Saliva was pre-absorbed with peptides immobilised on ELISA plates. Non-bound peptide specific IgA was then detected on plates coated with 5 μg/ml of individual peptides. This pre-absorption of saliva samples resulted in a reduction of PAB1.C7C-4 and -16 specific binding by approximately 50%. Pre-absorption with PAB1.C7C-3 did not reduce IgA reactivity. CarLA pre-absorption at 2 μg/ml was included as a positive control.

A subset of peptides that exhibited reactivity to salivary IgA and IgG was then used in absorption ELISAs. These assays were designed to test if reactivity could be reduced when the antibodies to CarLA, or to the peptide itself, were removed by pre-absorption. We found that pre-absorption of the saliva pool with PAB1.C7C-4 or PAB1.C7C-16, but not PAB1.C7C-3, reduced the reactivity to roughly 50% of the original reactivity, which was similar to what was seen for CarLA (Fig 4). This reduction in binding demonstrates selective depletion of peptide-specific salivary IgA, and thus provides additional evidence for the identification of biologically-relevant mimotopes of the PAB1 epitope.

**Fig 5:**
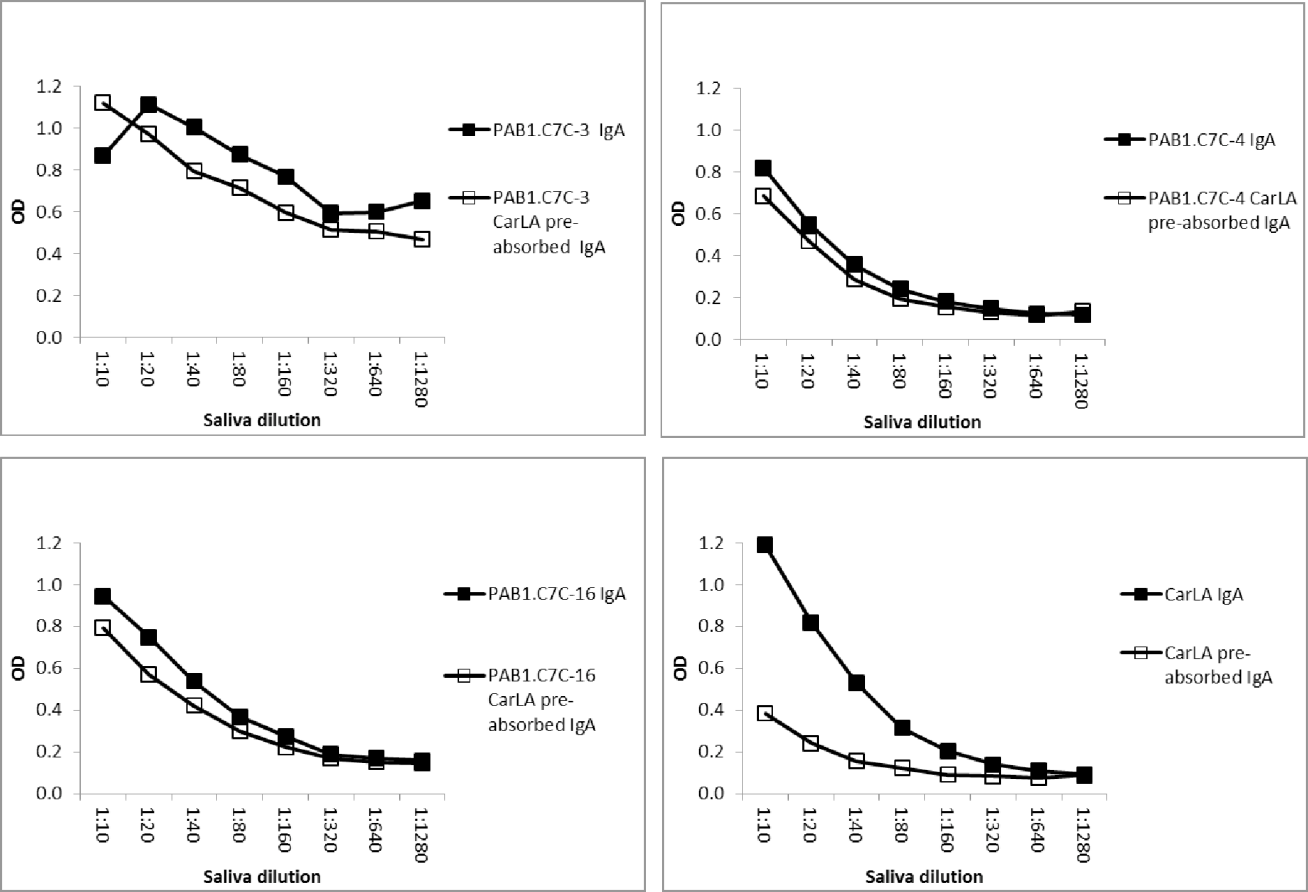
Pre-absorption of saliva with CarLA resulted in selective depletion of peptide specific IgA. Saliva was twice pre-absorbed with CarLA immobilised on ELISA plates. The saliva that was thus depleted of CarLA specific IgA was then tested for the ability to recognize individual peptides. This double pre-absorption of the saliva pool resulted in a detectable reduction of PAB1.C7C-3, -4 and -16 specific binding. Double pre-absorption with CarLA alone was included as a positive control.

**Fig 6:**
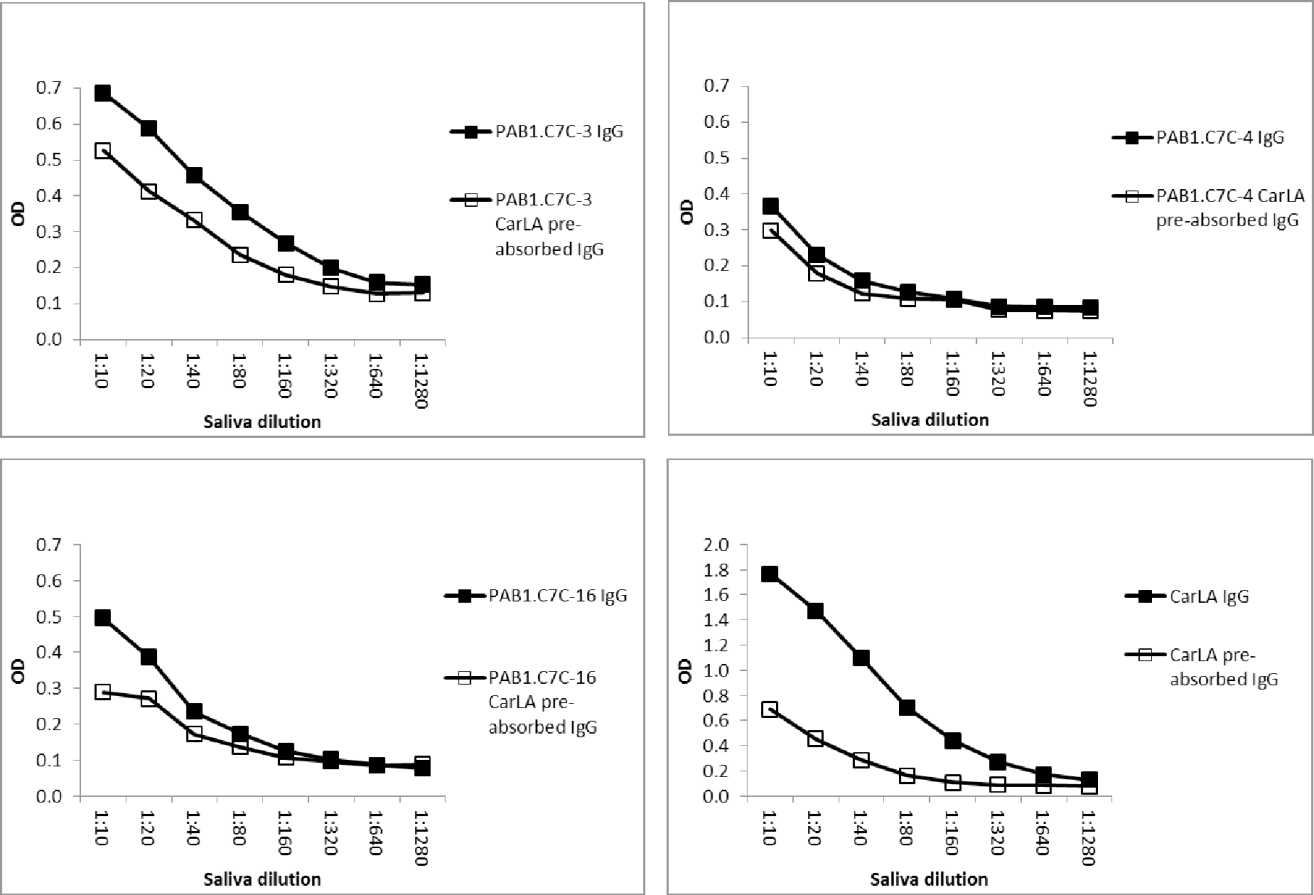
Pre-absorption of saliva with CarLA resulted in selective depletion of peptide specific IgG. Saliva was twice pre-absorbed with CarLA immobilised on ELISA plates. Non-bound CarLA specific IgG was then tested for the ability to recognize individual peptides. This double pre-absorption of samples resulted in a significant reduction of PAB1.C7C-3, -4, and -16 specific binding (P < 0.05). Double pre-absorption with CarLA alone was included as a positive control.

Furthermore, pre-absorption of saliva with CarLA resulted in a measurable reduction in antibody reactivity to individual peptides when the pre-absorption of the saliva pool was repeated a second time. This demonstrates that the reactivity to some of the peptides is part of the overall polyclonal salivary anti-CarLA specific IgA and IgG repertoire. A reduction in IgA-, but also of IgG-binding was observed for peptides PAB1.C7C-3, -4 and -16 (Figs 5 and 6, P< 0.05), and as expected to a greater extent for CarLA. Taken together, this set of saliva-absorption experiments provides strong additional evidence that a subset of the identified peptides are mimotopes of a CarLA epitope.

### CarLA binds to antibodies with higher affinity than peptide mimotopes

Individual peptide or a pool of peptides was tested for the ability to compete with immobilised CarLA for binding to the mAb PAB1. Neither individual nor peptide pools were able to inhibit binding of PAB1 to CarLA. Furthermore, when individual peptides were tested for the ability to compete with IgA from a CarLA-positive saliva pool, no reduction was found in binding of IgA to immobilised CarLA. This indicates that the affinities of polyclonal antibodies in saliva and of the mAb PAB1 for the native CarLA were higher than for the peptide mimotopes (data not shown).

### Serum antibodies raised against mimotopes react with the native parasite molecule CarLA

**Fig 7:**
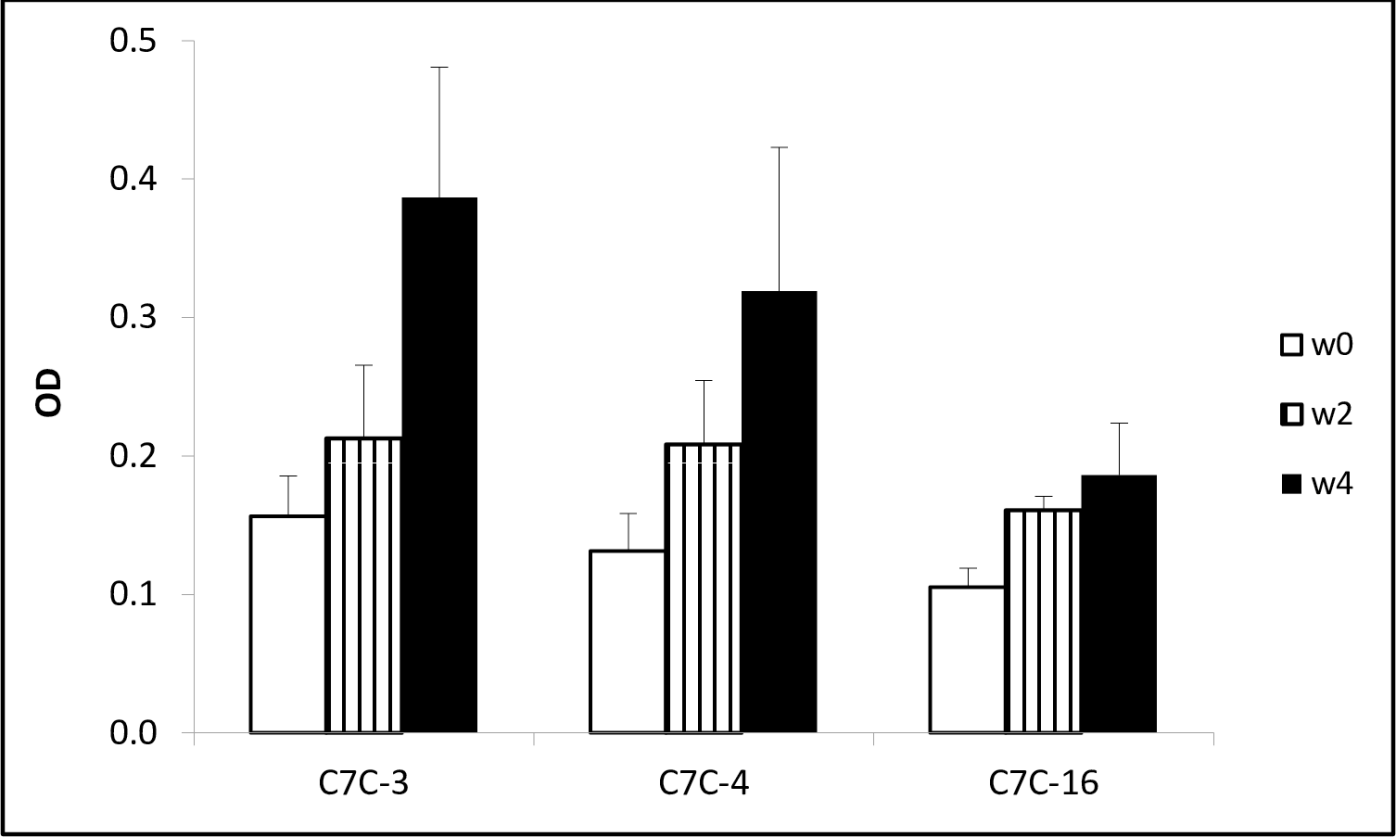
Vaccination against a mimotope induces an antibody response that recognises the native larval nematode glycan CarLA. Groups of mice (n = 3) were vaccinated twice at weeks 0 and 2 with individual phage clones PAB1.C7C-3, -4 or -16. Serum samples from weeks 0, 2 and 4 were analysed by ELISA for the presence of antibodies that bound to immobilised native CarLA, which revealed a significant increases (P < 0.05) in antibody levels following vaccination. Data are presented as means + SE of OD (n = 3).

Vaccinations against mimotopes were used to investigate if serum antibodies against the peptides react with the native carbohydrate larval antigen CarLA, which contains the epitope that is characterised by the mAb PAB1. We found that two vaccinations with the phage clones PAB1.C7C-3, -4 or -16 induced a serum antibody response that recognised the purified CarLA (Fig 7, P < 0.05). This confirmed that the peptides presented on selected phage clones were true mimotopes of the PAB1 epitope of CarLA.

## Discussion

In this study, we identified and characterised peptide mimotopes that share functional similarities with an epitope that is expressed on the nematode glycan CarLA, and which is recognised by the mAb PAB1. Antibodies in the saliva of nematode-immune animals that form part of the overall polyclonal mucosal immune response to CarLA specifically recognised these mimotopes. This demonstrates that antibodies to the PAB1 epitope are part of the anti-CarLA response. Furthermore, vaccinations against the mimotopes facilitated the induction of antibody responses that recognised the native molecule CarLA, demonstrating the biological relevance of these peptide mimics. These peptides are the first to mimic the PAB1 epitope of CarLA and thus form structural homologues of a defined glycan epitope which is conserved between many nematode species.

The nematode glycan CarLA is expressed at the infective larval stage of many livestock parasite species, and is shed from the surface during development to the L4 stage. Livestock exposed repeatedly to these gastro-intestinal parasites are able to develop a protective mucosal antibody repertoire, of which the anti-CarLA response forms a significant part [9]. Despite this, the immunological properties of CarLA are only partly known. Several epitopes implicated in protection have been described recently [4] but the conserved epitope recognised by the mAb PAB1 remains uncharacterised. Progress towards elucidating some of the functional properties has at least partly been hindered by a lack of structural information on CarLA, which results in the inability to synthesise the molecule. Peptide mimicry is increasingly used to identify and characterise important epitopes that consist of carbohydrate structures for viral, bacterial, and fungal pathogens, as well as for tumour antigens [5]. In this study we used peptide phage display technology to identify peptide mimotopes of the carbohydrate antigen CarLA. A disulphide-constrained peptide library was used for the selection process. These libraries are more likely to yield peptides that are superior to linear peptides in affinity [10], immunogenicity [11] and structural mimicry [12].

The ability to bind PAB1 to microbeads enabled efficient panning of the phage library. Several of the selected phage clones were repeatedly identified. This provided evidence of selective enrichment for binding to the target, although any amplification induced effects were not evaluated [13]. The ability to bind to PAB1 differed markedly between clones. Sequence analysis of the transduced amino acid sequences revealed three main clusters of peptides, containing peptide motifs TP and TTP, or SS, PS and SSP or SL. This suggests that these motifs are important for selection and form critical binding elements for specific IgA and IgG. As serine, threonine and proline are all neutral amino acids, and threonine and serine are both polar with alcohol based side chains, this may explain the mimicking of the binding sites by related amino acids to the surrogate carbohydrate epitope. Furthermore, it is likely that proline serves to constrain the conformations of the peptides, thereby exposing the serine and threonine to the antibody binding sites.

Synthetic peptides were tested for the ability to be recognised by polyclonal antibodies of IgA and IgG subclasses in the saliva of nematode-immune animals. Saliva was chosen over serum as it is the compartment that contains the antibody repertoire directly relevant to the prevention of establishment of incoming larvae, of which the anti-CarLA response forms a major part. We found that most peptides were recognised by antibodies in saliva to some degree, which provided additional evidence for the selection of biologically-relevant mimotopes, although their amino acid sequences differed. This is not too surprising as several glycomimetic cyclic peptides can exhibit similar physico-chemical properties to their surrogate carbohydrate molecule [14].

Further evidence for the biological relevance of the selected peptide mimotopes was obtained by pre-absorption of saliva with the native molecule CarLA. This resulted in a small but detectable reduction in IgA and IgG reactivity for PAB1.C7C-3, -4 and -16, which suggests that the polyclonal recognition of CarLA includes the reactivity to a part of the molecule that is mimicked by the peptide. Of note is that this reduction is smaller than that seen for CarLA. This indicates that the PAB1 epitope-specific antibodies are either over represented in the overall polyclonal antibody pool, and / or that the epitope is of low abundance on the molecule. This is supported by the fact that pre-absorption with peptides reduced the peptide specific component of the polyclonal saliva pool at similar levels as pre-absorption with CarLA. Interestingly, we did not detect binding of serum antibodies from nematode immune sheep to the selected peptides (data not shown). A reason for this inability to bind could be the presence of non-specified serum components that act as potent blocking agents for the specific antibody binding sites.

Mimotopes have been extensively used in vaccination studies to control a variety of human diseases including tumours and allergies [15, 16]. Mimotopes of carbohydrate antigens have also become essential tools in vaccine research since their discovery in the 1990s [17]. Furthermore, the immunogenic properties of mimotopes have also been exploited for research into controlling several parasitic diseases including infestations with *Trichinella spiralis* [18], *Rhipicephalus microplus* [19], *Fasciola hepatica* [20], *Taenia solium* [21] or *Schistosoma japonicum* [13]. We found that vaccinations with three specific phage clones induced an antibody response that reacted with the immobilised, purified surrogate carbohydrate molecule. Not only does this provide proof that the three peptides form true mimotopes of the nematode glycan CarLA, but also raises the potential for elucidating the biological function of the PAB1 epitope and its potential value in vaccination studies. It remains to be seen if mimotope vaccination is able to overcome significant obstacles due to the immunomodulatory properties of CarLA [22] and the generally poor immunogenicity of glycan molecules [17]. Future applications may also include their use in the diagnosis of animals that have developed protective immunity to nematode infection. Defined peptide antigens are already in use in the routine diagnosis of HIV infections [23] and are under development for human parasitic diseases such as neurocystocerciosis [24]. This, however, requires further study.

## Abbreviations

CarLA: carbohydrate larval antigen
PVP: Polyvinylpyrrolidone
TMB: Tetramethylbenzidine
